# Suggested Absence of Horizontal Transfer of Retrotransposons between Humans and Domestic Mammal Species

**DOI:** 10.3390/genes12081223

**Published:** 2021-08-08

**Authors:** Nicole M. Wanner, Christopher Faulk

**Affiliations:** 1 Department of Veterinary and Biomedical Sciences, College of Veterinary Medicine, University of Minnesota, 301 Veterinary Science Building, 1971 Commonwealth Avenue, St. Paul, MN 55108, USA; vbs@umn.edu; 2 Department of Animal Science, College of Food, Agriculture, and Natural Resource Sciences, University of Minnesota, 277 Coffey Hall, 1420 Eckles Avenue, St. Paul, MN 55108, USA

**Keywords:** transposons, horizontal transfer, transposomes, domestication

## Abstract

Transposable element sequences are usually vertically inherited but have also spread across taxa via horizontal transfer. Previous investigations of ancient horizontal transfer of transposons have compared consensus sequences, but this method resists detection of recent single or low copy number transfer events. The relationship between humans and domesticated animals represents an opportunity for potential horizontal transfer due to the consistent shared proximity and exposure to parasitic insects, which have been identified as plausible transfer vectors. The relatively short period of extended human–animal contact (tens of thousands of years or less) makes horizontal transfer of transposons between them unlikely. However, the availability of high-quality reference genomes allows individual element comparisons to detect low copy number events. Using pairwise all-versus-all megablast searches of the complete suite of retrotransposons of thirteen domestic animals against human, we searched a total of 27,949,823 individual TEs. Based on manual comparisons of stringently filtered BLAST search results for evidence of vertical inheritance, no plausible instances of HTT were identified. These results indicate that significant recent HTT between humans and domesticated animals has not occurred despite the close proximity, either due to the short timescale, inhospitable recipient genomes, a failure of vector activity, or other factors.

## 1. Introduction

Transposable elements (TEs) are DNA sequences that can move or copy themselves to new locations in genomes [1]. This property has led to TEs invading the genomes of nearly all living organisms to the point of being equal to or greater in quantity than host-derived sequences on a given chromosome [2]. TEs have strongly influenced genome structure and phenotypes in diverse organisms across evolutionary time via vertical inheritance from parent to offspring [3]. In addition to vertical inheritance, TEs can also be transmitted between unrelated individuals in the absence of reproduction through horizontal transfer (HT) [4,5,6]. The inherent mobilization properties of TEs enhance this phenomenon in eukaryotes, while HT of host genes is relatively rare [7,8], though the exact mechanisms dictating HT of TEs (HTT) are understudied. Generally, HTT is inferred by a higher than expected sequence identity between TEs across species compared to divergence based on vertical inheritance from the last common ancestor [9]. Both class I retrotransposons, which include long and short interspersed nuclear elements (LINEs and SINEs) and long terminal repeat (LTR) TEs, as well as class II DNA TEs, appear to be capable of HT between both near and distant lineages [10], although class II HTs are thought to be more prevalent [11]. HTT has also been found to impact gene expression and phenotype in plants and animals, lending an evolutionary pressure to transfer events [12,13,14]. Transfer of the P element in *Drosophila* was the first case of inferred HTT [15], with thousands of transfers having been subsequently identified in eukaryotes [12]. While HTT is generally more common in lower animals such as insects [16], evidence for the phenomenon has been found in domesticated mammal species. HT of the bovine-B (BovB) element has been inferred based on high sequence identity across genera and is present in cattle and horses [17,18]. Probable HTT vectors in animals include water, parasitic arthropods, and viruses [17,19].

Despite an increasing number of ancient HTT events being detected, studies evaluating evidence for more recent HTT are lacking. The use of consensus TE sequences when inferring HTT, while necessary for evaluating large-scale transfers across millions of years between many genomes, also prevents the detection of transfers currently in single or low copy numbers in the recipient genome. Human domestication of animals is a ready example of species sharing an environment conducive to HTT on a shorter timescale, on the order of tens of thousands of years or less [20]. Features favoring transmission include the fact that humans have shared close physical proximity with domesticated species through thousands of animal generations, infection by zoonotic pathogens, and cross-species parasitization by arthropods. Both humans and domesticated animals also experienced dramatic increases in population size as a result of their relationship, leading to increased opportunities for HTT. In contrast, the shared history of humans and domestic animals is exceedingly short on the scale of evolutionary time. This short time frame implies a low likelihood of HTT occurring, reaching the germline, and being vertically inherited widely enough to be present in most of the population. Linebreeding of domestic animals translates to a lower effective population size compared to wild animals [21]. Still, evidence for HTT between humans and domesticated animals has not been assessed in the literature, and inference for even single-copy HT on this timescale would have important implications for the ubiquity of HTT as a mechanism contributing to vertebrate evolution and phenotype.

The existence of high-quality reference genomes for humans and most domesticated animals finally makes the detection of low- and single-copy HTT events possible. Despite the close physical contact and other factors that make HTT more likely to be present, we expected the rarity of successful TE invasion and a lack of adequate time for any transferred TEs to become ubiquitous in the population to result in no detectable HTT events, even when searching millions of individual elements between human and domestic animal genomes. Here, we provide a comprehensive search for individual HTT events between humans and domestic animal species.

## 2. Materials and Methods

Reference genomes and retrotransposon genomic intervals (repeat classes LINE, SINE, and LTR) for human (GRCh38/hg38) and thirteen domestic animal species (dog, cat, horse, cow, pig, sheep, ferret, guinea pig, goat, alpaca, Bactrian camel, dromedary, and zebu) were downloaded from UCSC Table Browser using the RepeatMasker (rmsk) track (https://genome.ucsc.edu/ accessed on 30 May 2021). Only intervals longer than 100 bp were included. The search was also limited to retrotransposons based on a lack of evidence for significant DNA TE activity in mammals outside of the Vespertilionidae family of bats for a time period far exceeding the history of animal domestication by humans [22,23,24,25,26,27,28,29]. TableBrowser’s filter settings were also used to exclude tRNA intervals. TE sequences were extracted to multifasta files with the Biostrings package version 2.54.0 [30] in R version 3.6.1 (https://www.R-project.org/ accessed on 30 May 2021), and reference genomes downloaded from UCSC. TE intervals from non-chromosomal scaffolds were removed for species with chromosomal assemblies, while TE intervals were used in full for genomes without chromosome-level assemblies. A 400 bp decoy sequence (hg38 chr1:1000000–1000400) containing no TEs was appended to each retrotransposon multifasta; this sequence served as a positive control to verify that megablast was functional in the event of zero high-identity hits for a given pairwise comparison. Pairwise comparisons of reference mRNA sequences downloaded from UCSC were also performed as a positive control for vertical inheritance in a subset of domestic animal species.

Pairwise megablasts (either reference mRNA control or TE) were performed in the command line using BLAST+ version 2.8.1 [31]. The human suite of retrotransposons was made into a database object using the function makeblastdb, and the domestic animal multifastas were submitted individually as queries using the function blastn -task megablast. Minimum percent identity was set to 99%, e-value cutoff was set to 1e-25, and minimum query coverage (qcovs) was set to 95. These filtering criteria follow the assumption that any recently transferred TEs would have extremely high sequence identity in the host and recipient as dictated by a rate of mutation of 2.2 × 10^−9^ SNPs per base per year [32]. Using this mutation rate, we would expect a 1000 bp TE to collect less than 1 SNP in 100,000 years. High sequence identity and coverage expectations also dictated the use of megablast, which is recommended when the expected sequence identity is greater than 95%, rather than blastn. The same cutoff values were used for pairwise reference mRNA comparisons. Resulting megablast TE hits for each domestic animal species against human were assessed for vertical conservation manually in UCSC Genome Browser by comparing base level conservation in the Vertebrate Multiz Alignment & Conservation (100 Species) alignment track [33]. Vertical inheritance was determined under the assumption that horizontally transferred transposons would be absent in species more closely related to the target species. If hit coordinates showed TE conservation across at least 3 non-target species, then it was assumed to be the result of vertical transmission since TEs are homoplasy-free insertions whose ancestral state is the absence of the element [3].

## 3. Results and Discussion

Pairwise megablast queries of 27,949,823 retrotransposons from the reference genomes of humans and thirteen domestic animal species revealed zero insertion events consistent with HTT (Table 1). Initially, a total of 174 candidates were detected. Each species yielded between 4 and 21 hits to human passing identity (>99%) and coverage (>95%) stringency criteria, and the average hit length was 135 bp. The longest hit to human in any species was a 277 bp LINE fragment in pig, specifically a chicken repeat 1 (CR1) fragment, which had 99.6% sequence identity to a CR1 fragment in the human genome (Supplemental File 1). CR1s are ancient LINE elements found in amniotes, although the presence of CR1s in invertebrates has also been demonstrated [34,35]. In all, there were 121 LINE candidates with an average length of 135 bp, 50 candidate SINEs with an average length of 131 bp, and 3 LTR candidates with an average length of 182 bp (Supplemental File 1). This finding led to the elimination of LINE and LTR hits from the consideration for HTT, as transpositionally competent elements horizontally transferring between species in the last 10,000 years or less would be close to full length (>5000 bp) in both the source and target species. For successful expansion after invasion, a full-length LINE element is typically needed for functional retrotransposition. Evidence against HTT is strengthened by similarities in the number of hits and average hit length to pairwise queries of reference mRNA against human for a subset of species (Table 1).

Thirty-seven human TEs also yielded high-identity hits in two or more domestic animal species (Supplemental File 2); high identity in multiple species provides evidence for vertical inheritance, and these hits were therefore eliminated as HTT candidates. In a case of HT, we expect extremely high identity between the host and target species and lower coincidental identity of unrelated copies in other animals, or the absence of the element depending on the direction of transfer. LINE fragments were the most common candidates detected, as expected from their ancient and ubiquitous spread among chordates. HT of LINEs is also thought to occur with some frequency, with examples including BovB elements transferred to ruminants from squamates and L1s and species-specific LINEs in marine eukaryotes [9,17,47,48]. The ubiquity of LINEs in vertebrates and their ability to independently produce their own retrotransposition machinery make them excellent candidates for HTT. Despite this, we found no evidence of LINE HT between humans and domestic species. Further evidence against HTT was found in the type of elements passing stringency criteria; common hits included CR1 LINE fragments, MIR SINEs, and AmnSINE1s, all of which are ancient elements in the amniote lineage, suggesting false positives based on highly conserved sequence similarity [22,49,50]. Confirmation of ancient vertical transmission was evident in their presence in at least three additional species in syntenic regions.

**Figure 1:**
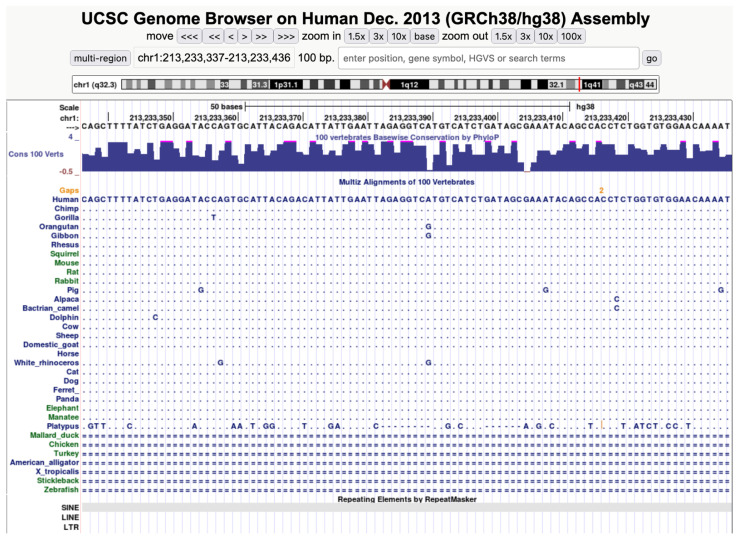
Example of evidence for ancient vertical conservation of a high-identity human vs. domestic animal SINE hit in UCSC Genome Browser. The human sequence is represented horizontally by base pair, while alignments for various domestic and wild animal species are matched by base pair position below; dots indicate conserved nucleotides, while SNPs are shown as the modified base against human. Dashes indicate gaps (absence or truncation of the element). A horizontally transferred element would have extremely high sequence similarity in human and the target species while having a lower identity in species related to the host and being absent in species related to the target. Accessed on 30 July 2021.

For the eight SINE hits initially detected as unique to human and one other species, manual inspection with the 100 Species MultiZ alignment track in UCSC Genome Browser revealed strong conservation across multiple vertebrate genera and orders (Figure 1), providing evidence for vertical rather than horizontal inheritance of the elements. While SINEs depend on other TEs for their retrotransposition, evidence of SINE HT is present in the literature. The relatively young 5S RNA-derived SINE PxSE4 shows evidence of HT in lepidopteran insects [51], and novel HaSE SINEs show signatures of HT in the cotton bollworm, *Helicoverpa armigera* [52]. Indicators of HT have also been found for a SINE shared by reptiles and mammals and the SmaI SINE family in salmonid fishes [53,54]. While the high copy number and small size of SINEs make them good HT candidates in theory, retrotransposon HT is generally much rarer than DNA TE transfer, likely due, in part, to the inherent instability of retrotransposon RNA intermediates.

The lack of HTT between humans and domestic animal species was consistent with the study expectations. We chose domestic species due to humanity’s history of unnaturally close proximity, shared parasitization by plausible HTT vectors (which have served as HTT vectors in other clades), and rapid expansion of human and domestic animal population sizes. An illustrative case is the BovB element, which has proliferated rapidly in species as divergent as bovines and squamates and ultimately makes up 12% of the cattle genome [18]. However, the tick vector that likely transferred BovB has been active for tens of millions of years, many orders of magnitude longer than humans have coexisted with domestic species. Animal domestication estimates indicate that humans and most of the queried species (with the exception of dogs, which were likely domesticated before the advent of agriculture [36]) have coexisted in close proximity for only 2000 to 11,000 years [20]. These short periods of contact translate to a low likelihood of a germline HT event occurring and vertically propagating through populations to be present in reference human or animal genomes. In the event that horizontally transferred sequences did reach a new organism in this time frame, incompatibility with the target genome’s transcriptional machinery is another hurdle to successful incorporation, particularly for retrotransposons [55,56].

Our search demonstrates a lack of evidence for recent HTT between humans and domestic species. Prior identification of HTT events used consensus sequences of transposons derived from multiple paralogs within a species, compared to consensus or individual insertions in an unrelated species. The advantage of our study is in the ability to detect HTT resulting in few or even single insertion events. Still, the use of reference genomes allows for the possibility of HTT detection failure due to population heterogeneity, somatic HT, inadequate genome coverage or errors (particularly over repetitive regions) or other factors. However, the lack of plausible horizontally transferred elements within human and animal reference genomes provides adequate evidence to assume that the phenomenon is not common or widespread.

## Figures and Tables

**Table 1:** Summary values for human vs. domesticated animal pairwise megablasts performed to identify signatures of recent retrotransposon HT. Pairwise megablasts of reference mRNA were performed for a subset of species as a positive control for vertical inheritance. TE: transposable element; kya: thousands of years ago.

Animal	Genome	Approximate Domestication (kya)	*n* Queried (mRNA)	*n* Hits (mRNA)	Mean Hit Length (mRNA)	*n* Queried (TE)	*n* Hits (TE)	Mean Hit Length (TE)
Human	hg38					3,402,790		
Dog	canFam5	12–33 [36]				1,748,840	19	138
Cow	bosTau9	>10 [37]	14,530	132	220	3,117,396	12	135
Horse	equCab3	5.5 [38]	1878	117	228	2,034,000	21	128
Sheep	oviAri4	11 [39]	1018	25	85.2	2,960,232	9	135
Pig	susScr11	9 [20]	4851	98	90	1,789,659	13	144
Guinea pig	cavPor3	5 [40]	491	51	121	1,721,346	5	122
Cat	felCat9	4 [41]	419	0		1,855,102	20	138
Alpaca	vicPac2	7 [42]	17	0		1,739,851	18	140
Ferret	musFur1	2 [43]				2,545,632	4	125
Bactrian camel	GCF_000767855.1	5 [44]				1,586,346	15	134
Dromedary	GCF_000803125.2	4 [45]				1,663,540	18	134
Goat	GCF_001704425.1	11 [39]				2,153,565	9	133
Zebu	GCF_000247795.1	8 [46]				3,034,305	11	130

## Data Availability

Publicly available datasets were analyzed in this study. These data can be found here: https://genome.ucsc.edu/; accessed on 4 June 2021.

## Supplementary Materials

The following are available online at https://www.mdpi.com/article/10.3390/genes12081223/s1, Supplemental File 1: All high-stringency pairwise BLAST results for individual transposable elements between human (hg38) and domestic animal species. Supplemental File 2: Summary table for TE hits to human found in multiple domesticated animal species. Chromosomal locations and names are derived from the human genome (hg38).
